# Prostaglandin E_2_ Impairs P2Y_2_/P2Y_4_ Receptor Signaling in Cerebellar Astrocytes via EP3 Receptors

**DOI:** 10.3389/fphar.2017.00937

**Published:** 2017-12-22

**Authors:** Lucía Paniagua-Herranz, Juan C. Gil-Redondo, Ma José Queipo, Silvia González-Ramos, Lisardo Boscá, Raquel Pérez-Sen, Ma Teresa Miras-Portugal, Esmerilda G. Delicado

**Affiliations:** Departamento de Bioquímica y Biología Molecular IV, Facultad de Veterinaria, Instituto Universitario de Investigación en Neuroquímica, Instituto de Investigación Sanitaria del Hospital Clínico San Carlos, Universidad Complutense Madrid, Madrid, Spain

**Keywords:** astrocytes, calcium, EP receptors, nucleotide receptor, P2Y receptors, PGE_2_

## Abstract

Prostaglandin E_2_ (PGE_2_) is an important bioactive lipid that accumulates after tissue damage or inflammation due to the rapid expression of cyclooxygenase 2. PGE_2_ activates specific G-protein coupled EP receptors and it mediates pro- or anti-inflammatory actions depending on the cell-context. Nucleotides can also be released in these situations and they even contribute to PGE_2_ production. We previously described the selective impairment of P2Y nucleotide signaling by PGE_2_ in macrophages and fibroblasts, an effect independent of prostaglandin receptors but that involved protein kinase C (PKC) and protein kinase D (PKD) activation. Considering that macrophages and fibroblasts influence inflammatory responses and tissue remodeling, a similar mechanism involving P2Y signaling could occur in astrocytes in response to neuroinflammation and brain repair. We analyzed here the modulation of cellular responses involving P2Y_2_/P2Y_4_ receptors by PGE_2_ in rat cerebellar astrocytes. We demonstrate that PGE_2_ inhibits intracellular calcium responses elicited by UTP in individual cells and that inhibiting this P2Y signaling impairs the astrocyte migration elicited by this nucleotide. Activation of EP3 receptors by PGE_2_ not only impairs the calcium responses but also, the extracellular regulated kinases (ERK) and Akt phosphorylation induced by UTP. However, PGE_2_ requires epidermal growth factor receptor (EGFR) transactivation in order to dampen P2Y signaling. In addition, these effects of PGE_2_ also occur in a pro-inflammatory context, as evident in astrocytes stimulated with bacterial lipopolysaccharide (LPS). While we continue to investigate the intracellular mechanisms responsible for the inhibition of UTP responses, the involvement of novel PKC and PKD in cerebellar astrocytes cannot be excluded, kinases that could promote the internalization of P2Y receptors in fibroblasts.

## Introduction

Prostaglandins are bioactive lipids produced through the metabolism of arachidonic acid by cyclooxygenase and they play important modulatory roles throughout the body (Sugimoto and Narumiya, 2007). Both COX-1 and COX-2 display cyclooxygenase activity, which are constitutive and inducible enzymes, respectively (Smith et al., 2000). These enzymes convert the arachidonic acid released following phospholipase A_2_ (PLA_2_) activation from membrane lipids into PGH_2_, the precursor of both PGs and thromboxanes. One of the principal activators of PLA_2_ is intracellular calcium, which brings arachidonic acid production under the control of multiple extracellular signals, including extracellular nucleotides (Zonta et al., 2003). PGE_2_ is one of the most studied PGs, and not only is it the most widely found in different animal species (including humans) but it also exhibits the most versatile actions. Through specific membrane receptors, PGE_2_ fulfills many physiological functions, such as gastrointestinal mucosa protection or labor, as well as participating in pathological events like inflammation or fever. Indeed, the cyclooxygenase enzymes have been widely explored in anti-inflammatory therapies, although the majority of COX inhibitors display side effects due to COX-1 inhibition, mainly gastrointestinal bleeding and renal toxicity (Dannhardt and Kiefer, 2001; Dwivedi et al., 2015).

Prostaglandin E_2_ activates specific GPCRs, named EP receptors. Four different EP receptor subtypes have been identified, EP1–EP4, which differ in the signal transduction pathways they use, their distribution and the regulation of their expression (St-Jacques and Ma, 2016). The EP1 receptor is mainly coupled to Gq proteins and its stimulation induces intracellular calcium responses. By contrast, the EP2 and EP4 receptors are coupled to Gs proteins and they produce an accumulation of cAMP, whereas the EP3 receptor is mainly coupled to Gi proteins and it provokes a decrease in the cAMP produced in response to Gs-protein coupled receptor stimulation. There are three EP3 splice variants (α, β, and γ), each of which contains a distinct C-terminal tail of 30, 26, and 29 amino acids, respectively. These isoforms all have similar ligand binding properties but they activate different signal transduction pathways. Indeed, the EP3 variants not only couple to Gi proteins but also, to Gq proteins, Rho or even adenylate cyclase. In addition, some effects of PGE_2_ are exert independently of EP receptors and other intracellular targets have even been proposed (Traves et al., 2013).

Extracellular nucleotides are also important extracellular messengers (Burnstock, 2006; Miras-Portugal et al., 2016). Nucleotides can be released from living cells under basal conditions and their release is enhanced by cell stressors, including mechanical stress, hypoxia, viral infection or pro-apoptotic stimuli. Nucleotides are stored in secretory vesicles along with biogenic amines and other neurotransmitters, and they are released by exocytosis (Lazarowski et al., 2011). Once in the extracellular space, nucleotides interact with specific membrane nucleotide receptors, which are classified in two main types: the ionotropic P2X receptors and the metabotropic P2Y receptors. Seven P2X nucleotide receptors have been identified and they are trimeric ATP-activated ion channels that are permeable to Na^+^, K^+^, and Ca^2+^ (North, 2016). Metabotropic P2Y receptors belong to the superfamily of GPCRs and unlike P2X receptors, they can be activated by adenine and/or uridine nucleotides (ATP, ADP, UTP, and UDP) and nucleotide sugars (UDP-glucose). Eight subtypes of P2Y receptors have been identified that have been classified in two main subfamilies according to their pharmacology, signal transduction and structure (Costanzi et al., 2004; von Kugelgen and Hoffmann, 2016). The first of these is coupled to Gq proteins and phospholipase C (PLC), and it includes the P2Y_1_, P2Y_2_, P2Y_4_, P2Y_6_, and P2Y_11_ receptors. By contrast, the second one is linked to Gi/Go proteins and it includes the P2Y_12_, P2Y_13_, and P2Y_14_ receptors. However, like other GPCRs, P2Y receptors are promiscuous and additional G protein linkages have been described (Erb and Weisman, 2012). P2X and P2Y receptors are co-expressed throughout the body, and they play important roles in the vascular system, in immune defense and in the nervous system. The extracellular concentration of nucleotides is controlled by a family of ectoenzymes that catalyze the hydrolysis of nucleotides, generating their corresponding diphosphate nucleotides and ultimately, nucleosides. These nucleosides are then taken back up by the cells via nucleoside transporters.

Both, PGE_2_ and nucleotides have beneficial or detrimental effects depending on the cell context, and the duration of PGE_2_ production or nucleotide release. Furthermore, they can co-exist in the extracellular milieu under physiological and pathological conditions. As mentioned previously, nucleotides can influence PGE_2_ production and *vice versa*, PGE_2_ may regulate nucleotide activity (Chen and Lin, 2000; Zonta et al., 2003; Ito and Matsuoka, 2008, 2015). In this regard, we previously showed that PGE_2_ inhibited P2Y responses in macrophages, particularly those mediated by P2Y receptors sensitive to purine and pyrimidine nucleotides, the metabotropic P2Y_2_, P2Y_4_, and P2Y_6_ receptors. The selective impairment of P2Y signaling by PGE_2_ appears to be independent of EP receptors, involving the activation of nPKC and PKD, and with functional implications for the biological responses of macrophages due to the influence on their metabolism and migration. Such interactions take place in thioglycollate-elicited and alternative macrophages, which could contribute to the resolution phase of inflammation (Traves et al., 2013). The same cross-talk between PGE_2_ and P2Y signaling has been described in murine fibroblasts, suggesting that macrophages and fibroblasts contribute to the regulation of inflammatory responses and tissue damage repair through aligned mechanisms associated with P2Y signaling. Such cross-talk may open new avenues to develop future anti-inflammatory therapies (Pimentel-Santillana et al., 2014).

In an attempt to investigate whether PGE_2_ would modulate nucleotide P2Y responses in the nervous system we decided to analyze this interaction in astrocytes. Thanks to their strategic location in close proximity with neurons and endothelial cells, astrocytes regulate cerebral blood flow to metabolic demands, PGE_2_ being one of the first described mediators of cerebral vasodilation (Ellis et al., 1979; Takano et al., 2006). Astrocytes are also able to detect danger signals, secrete cytokines, and activate adaptive defense [see excellent reviews (Sofroniew, 2015; Colombo and Farina, 2016)]. Moreover, the typical hallmark of brain injury is the upregulation of the astrocyte marker, the glial fibrillary acidic protein (GFAP), as consequence of astrocyte proliferation and the glial scar formation. The impact of astrocytes activity on tissue homeostasis is ambivalent, it may exacerbate inflammatory responses and tissue damage, or promote immunosuppression and tissue repair, depending on timing and context. *In vivo* studies performed in disease models of brain or spinal cord injury and experimental autoimmune encephalomyelitis (EAE) have revealed that the loss of reactive astrocytes during the early phases of injury results in exacerbation of clinical signs, motor deficits, scar disorganization, demyelination and neuronal death. By contrast, astrocyte depletion during chronic phase of EAE ameliorates disease and reduces leukocyte infiltration into nervous system. Recently, it has been described that astrocytes also respond to proinflammatory stimuli with the intense production of PGE_2_, adapting cerebral blood flow to neuro-inflammatory demands (Font-Nieves et al., 2012; Howarth et al., 2017).

Astrocytes represent a heterogeneous cell population in terms of their ability to respond to neuroligands linked to calcium mobilization and they are also able to selectively discriminate between the activities of different pathways that use the same neurotransmitter. However, native and cultured astrocytes from different brain areas, cortical, hippocampal and cerebellar astrocytes are sensitive to nucleotides given that they co-express P2Y and P2X nucleotide receptors (Neary et al., 2004; Domercq et al., 2006; Fujita et al., 2009; Peterson et al., 2010; Oliveira et al., 2011). The present study was performed on cultured cerebellar astrocytes, because they constitute a homogeneous cell population that exhibited functional P2Y nucleotide receptors, especially those we are interested, the P2Y_2_ and P2Y_4_ receptors sensitive to adenine and pyrimidine nucleotide (ATP/UTP). In previous studies we demonstrated that metabotropic ATP calcium responses were submitted to a fine regulation by growth factors (EGF/NGF) and signals coupled to Gs stimulation, such as noradrenaline and adenosine, which would suggest the importance to maintain purinergic tone. Using different approaches we found that PGE_2_ inhibits P2Y_2_/P2Y_4_ signaling in rat cerebellar astrocytes. Indeed, PGE_2_ reduced UTP calcium responses and intracellular signaling cascades activated by this nucleotide, as well as preventing UTP-induced cell migration. In astrocytes, the PGE_2_ effect appeared to be mediated by EP3 receptors and was also observed in LPS-treated cells.

## Materials and Methods

### Chemicals, Materials, and Antibodies

Papain was purchased from Worthington (Lake Wood, NJ, United States), FCS from Invitrogen and Fura-2 from Molecular Probes (Life Technologies, Barcelona, Spain). Culture flasks, Plastic Petri dishes and transwell chambers with 8 μm pore transparent PET membrane inserts were supplied by Falcon Becton Dickinson Labware (Franklin Lakes, NJ, United States). Antibiotics, DMEM, UTP, and AG1478 were purchased from Sigma Aldrich (St. Louis, MO, United States). PGE_2_, and the EP agonists and antagonists, were from Cayman Chemical (Ann Arbor, MI, United States), while LY294002 was from Calbiochem Co. (San Diego, CA, United States). Specific antibodies against phosphor-ERK1/2 (Tyr^204^), ERK2, phosphor-Akt (Thr^308^) and Akt were purchased from Santa Cruz Biotechnology (Santa Cruz, CA, United States), and anti-COX-2 was from Abcam (Cambridge, United Kingdom). Antibodies against P2Y receptors, P2Y_2_ and P2Y_4_ receptors, and those against the EP3 receptor were from Alomone Labs (Jerusalem BioPark, Israel), while antibodies against GAPDH were purchased from Cell Signaling Technology (Beverly, MA, United States). Secondary horseradish peroxidase-conjugated anti-mouse and anti-rabbit antibodies (Dako, Denmark) were used here. All other reagents not specified were routinely supplied by Sigma, Merck (Darmstadt, Germany) or Roche Diagnostics SL (Barcelona, Spain).

### Experimental Animals

All the experiments were carried out at the Complutense University of Madrid (Madrid, Spain) following the International Council for Laboratory Animal Science guidelines (ICLAS). All the procedures were approved by both the Animal Ethics Committee of the Complutense University and the Regional Authorities in Madrid (Area of Animal Protection), according to the Spanish Royal Decree RD53/2013 and the European Directive 2010/63/UE on the protection of animals used for scientific purposes. The assays were designed to minimize the number of animals used while maintaining statistical validity.

### Astrocyte Cultures

Primary cultures of astrocytes were prepared as described previously with some modifications (Jimenez et al., 1999). Briefly, the cerebellum from Wistar rat pups (P7) was removed aseptically, digested with papain and cerebellar cells were resuspended in DMEM containing 10% FCS (v/v), 25 mM glucose, 2 mM glutamine, 100 U/ml penicillin, 100 μg/ml streptomycin, and 2.5 μg/ml amphotericin. The cells were plated in culture flasks at density of 70,000 cells/cm^2^ and they were maintained in culture until reaching confluence (approximately 10–12 days), replacing the medium every 3–4 days. Cultures were depleted of microglial cells by orbital shaking and finally, the astrocytes were detached from the culture flasks by trypsin treatment and seeded onto culture plates. For microfluorometry experiments, astrocytes were plated onto glass coverslips in 35 mm Petri dishes at 50,000 cells/cm^2^ and for Western blotting, cells were plated onto Petri dishes at a density of 35,000 cells/cm^2^. Astrocytes were routinely used 48 h after plating.

### Fura-2 Microfluorimetry and Calcium Imaging

Calcium imaging experiments were carried out essentially as described previously (Carrasquero et al., 2005). Astrocytes attached to coverslips were incubated in Locke’s solution (composition in mM: 140 NaCl, 4.5 KCl, 2.5 CaCl_2_, 1.2 KH_2_PO_4_, 1.2 MgSO_4_, 5.5 glucose, 10 HEPES pH 7.4) supplemented with 1 mg/mL bovine serum albumin (BSA) and loaded with 5–7 μM fura-2/AM for 45 min at 37°C. Once washed in fresh Locke’s solution, a coverslip was placed in a small superfusion chamber and the cells were imaged on a NIKON TE-200 microscope equipped with a Plan Fluor 20X/0.5 objective. Cells were continuously superfused with Locke’s medium at a rate of 1.5 mL/min, and agonists were usually applied for 20 s by switching the superfusion solution with the aid of WC-8 valve controller (Warner Instruments). Cells were illuminated at 340 and 380 nm, and the emitted light was isolated with a dichroic mirror (430 nm) and a 510 nm band-pass filter (Omega Optical). Images were obtained with an ORCA-ER C 47 42-80 camera (Hamamatsu City, Japan) controlled by MetaFluor 6.2r and PC software (Universal Imaging, Corp., Cambridge, United Kingdom). The exposure time was 100 ms and the wavelength of incoming light was changed from 340 to 380 nm in less than 5 ms. Fluorescence images were acquired at a sampling frequency of 2 Hz and processed by averaging signals from small elliptical regions within individual cells. Background signals were subtracted from each wavelength and the F_340_/F_380_ fluorescence ratio was calculated. Fluorescence ratio increases represent [Ca^2+^]_i_ changes. The quantification of the responses was made by measuring the magnitude of the initial transients. Data are represented as the percentage of the control responses elicited by an UTP challenge in the same experimental conditions (each individual cell).

### Migration Assays

Astrocytes were detached from the culture flasks and they were suspended in DMEM supplemented with 1% FCS. The lower chambers of Transwell 6-well culture plates were filled with 3 mL DMEM containing UTP (as indicated), and with an 8 μm membrane insert placed into the wells, a 1 mL cell suspension containing 300,000 cells was added to the upper chamber. After 18 h at 37°C in a 5% CO_2_ incubator, the cells in the upper chambers were removed by scraping the membranes with a cotton swab, while the cells attached to the bottom of the membranes were fixed with 4% paraformaldehyde for 15 min, washed with PBS and stained with the nuclear marker, DAPI. The cells were visualized under a microscope and counted in five random fields. They cells attached to the bottom of the membrane represented those that had migrated from the upper to lower chamber in response to nucleotide stimulation.

### *In Vitro* Wound Healing

Astrocytes detached from culture flasks were seeded onto Petri dishes in complete culture medium and they were maintained in the incubator to form a confluent monolayer (48 h after plating). A wound was made by scraping a conventional yellow pipette tip across the monolayer and the cells were then washed with culture medium, replacing the medium with fresh DMEM plus 1% FCS and with the addition of the nucleotide or another effector to induce migration. After 24 h at 37°C in a 5% CO_2_ incubator, the cells were fixed with paraformaldehyde as described above and their nucleus was stained with DAPI.

### Cell Treatments and Lysate Preparations

Cells were stimulated in Locke’s solution and where indicated, the astrocytes were preincubated in the presence or absence of effectors, antagonists or inhibitors prior to stimulation with the nucleotide UTP. Controls were systematically used with the corresponding vehicle alone (DMSO always at a concentration <2%), in which cell viability was not significantly affected. Incubations were stopped by removing the incubation medium and lysing the cells in cold lysis buffer (20 mM MOPS pH 7.2, 50 mM NaF, 40 mM β-glycerophosphate, 1 mM sodium orthovanadate, 5 mM EDTA, 2 mM EGTA, 0.5% Triton X-100, 1 mM PMSF, and a protease inhibitor cocktail).

### Western Blotting

Total cellular lysates (15–30 μg protein) were resolved in SDS–PAGE gels and transferred to PVDF membranes. The membranes were probed overnight at 4°C with primary antibodies in either TBS 0.1% with Tween-20 containing low-fat milk powder (5%, w/v), and then for 1 h at room temperature with the secondary antibodies. Antibodies were used at following dilutions: anti-phosphor-ERK1/2, anti-ERK2 (1:1000), anti-phosphor Akt and Akt (1:1000), anti-COX-2 (1:500), anti-P2Y_2_ and anti-P2Y_4_ (1:200), anti-EP3 receptors (1:200), and anti-mouse and anti-rabbit horseradish peroxidase-conjugated secondary antibodies (1:2000 and 1:1000, respectively). Specific protein bands were visualized by ECL (Western Lighting ECL PRO kit, Perkin Elmer, Madrid, Spain), and chemiluminescence images were taken with the ImageQuant LAS 500^®^ image system and quantified by densitometry using ImageQuantTL software.

### Statistical Analysis

The results are expressed as the means ± SEM calculated from at least three experiments performed on cells from different cultures. When multiple comparisons were made, one-way analysis of variance was used and Dunnett’s post-test analysis was applied only when a significant (*p* < 0.05) effect was indicated by the one-way analysis of variance (GraphPad Prism 5; GraphPad Software, Inc., San Diego, CA, United States).

## Results

### PGE_2_ Modulates Metabotropic ATP Calcium Responses in Individual Cerebellar Astrocytes

**FIGURE 1:**
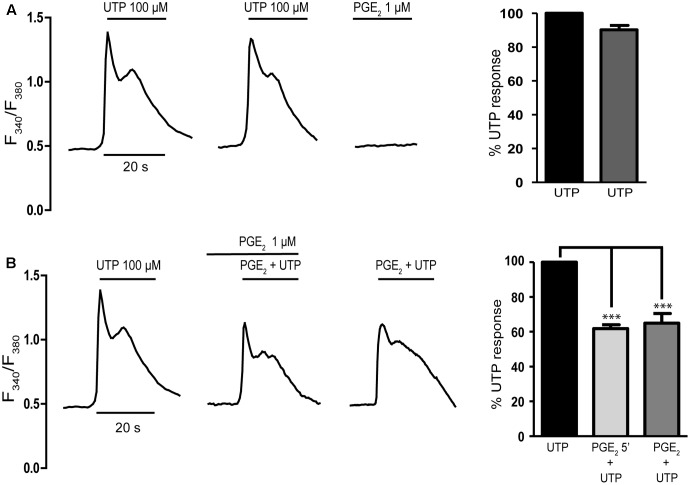
Prostaglandin E_2_ (PGE2) inhibits UTP calcium responses in rat cerebellar astrocytes. **(A)** Typical traces of calcium responses induced by 100 μM UTP in individual astrocytes. Cells loaded with fura-2 were challenged with the nucleotide or PGE_2_ (1 μM) and calcium responses were recorded as described in the Section “Materials and Methods.” **(B)** Effect of PGE_2_ on UTP calcium responses. Astrocytes were incubated in the presence or absence of PGE_2_ (1 μM) and subsequently stimulated with UTP (100 μM), again in the presence or absence of PGE_2_. Typical recordings are shown and the graphs show the quantification of the responses, obtained by measuring the magnitude of the initial transients. The data are presented as the percentage of the control responses elicited by UTP and the values are the means ± SEM (*n* = 327 cells from four different cultures). ^∗∗∗^*p* < 0.0001 according to one way analysis of variance and Dunnett’s post-test.

Cerebellar astrocytes co-express several subtypes of P2Y metabotropic nucleotide receptors (Jimenez et al., 2000), and metabotropic calcium responses to ATP mediated by P2Y_2_/P2Y_4_ nucleotide receptors were evident in the entire astrocyte population, in which ATP and UTP were equipotent. To examine the modulatory role of PGE_2_ on P2Y signaling in cerebellar astrocytes, we investigated the effect of this prostaglandin on UTP induced calcium responses. We did not use ATP, because cerebellar astrocytes also express ATP sensitive P2X7 receptors (Carrasquero et al., 2009). As expected all the cells tested responded to the UTP challenge (100 μM) by increasing their intracellular calcium [Ca^2+^]_i_, which peaked (ΔF_340_/F_380_ of 0.73 ± 0.02 relative to the basal levels between 0.3 and 0.5; *n* = 327 cells) and then returned to the basal level within the first 20 s of stimulation (**Figure 1A**). These cells also responded to a second UTP challenge with similar calcium transients, the magnitude of which was reduced by 10%. It is known that EP1 receptors couple to Gq proteins and EP2 receptor stimulation promotes calcium responses in rat cortical astrocytes (Di Cesare et al., 2006). Thus, before analyzing the modulatory role of PGE_2_ on UTP calcium responses, cells were challenged with PGE_2_ (1 μM) alone, which did not evoke any increase in [Ca^2+^]_i_. Cells were also insensitive to stimulation with 6,16-Dimethylprostaglandin E_2_, a synthetic analog that resists metabolism and that has a prolonged half-life *in vivo* (results not shown). These results indicated that cerebellar astrocytes did not express functional EP1 receptors, although they did not rule out the presence of functional EP2/EP4 receptors coupled to adenylate cyclase activation. PGE_2_ inhibited UTP calcium responses (**Figure 1B**) and preincubation with PGE_2_ (1 μM, 5 min) dampened the calcium responses elicited by 100 μM UTP, the maximal effective concentration. This inhibition was observed in all the cells tested and the magnitude of the initial Ca^2+^ transient was reduced by a 40%. Nevertheless, the effect of preincubation with PGE_2_ was not necessary due to the simultaneous co-stimulation of astrocytes with PGE_2_ and UTP reduced the calcium responses triggered by the nucleotide alone by 35%. These results were consistent with the regulatory influence of PGE_2_ on P2Y signaling evident in macrophages and fibroblasts (Ito and Matsuoka, 2008; Traves et al., 2013; Pimentel-Santillana et al., 2014).

**FIGURE 2:**
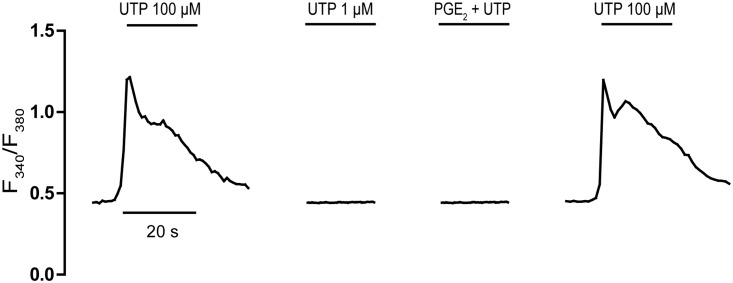
Effect of PGE_2_ on an ineffective UTP challenge. Astrocytes were stimulated with UTP (100 μM) or an ineffective concentration of UTP (1 μM), alone or in conjunction with PGE_2_ (1 μM), and the calcium responses were recorded as described in Section “Materials and Methods.”

The presence of EP2/EP4 receptors coupled to Gs proteins in cerebellar astrocytes was analyzed indirectly, assessing whether UTP-mediated responses could be enhanced by PGE_2_ (**Figure 2**). We previously reported an important cross-talk between Gs-coupled receptors and P2Y nucleotide receptors in these glial cells (Jimenez et al., 1999). Indeed, the activation of A_2B_ adenosine receptors, or other signals coupled to adenylate cyclase stimulation, strongly potentiated the metabotropic calcium responses to nucleotides. Co-stimulation of astrocytes with adenosine (10 μM) and ATP (1 μM, ineffective when administered alone) elicited [Ca^2+^]_i_ transients that represented 60% of the maximal calcium response elicited by ATP/UTP (100 μM). This potentiation was parallel to but independent of cAMP accumulation, suggesting the involvement of βγ subunits released upon Gs stimulation. Co-stimulation of astrocytes with PGE_2_ and ineffective concentrations of UTP (1 μM) did not evoke any signals (**Figure 2**), and no signal was observed after preincubation with PGE_2_ and simultaneous co-stimulation with UTP (1 μM). Hence, cerebellar astrocytes do not appear to express functional EP2/EP4 receptors and if they are present, they were unable to modulate UTP calcium responses in the same way as other signals coupled to adenylate cyclase activation.

**FIGURE 3:**
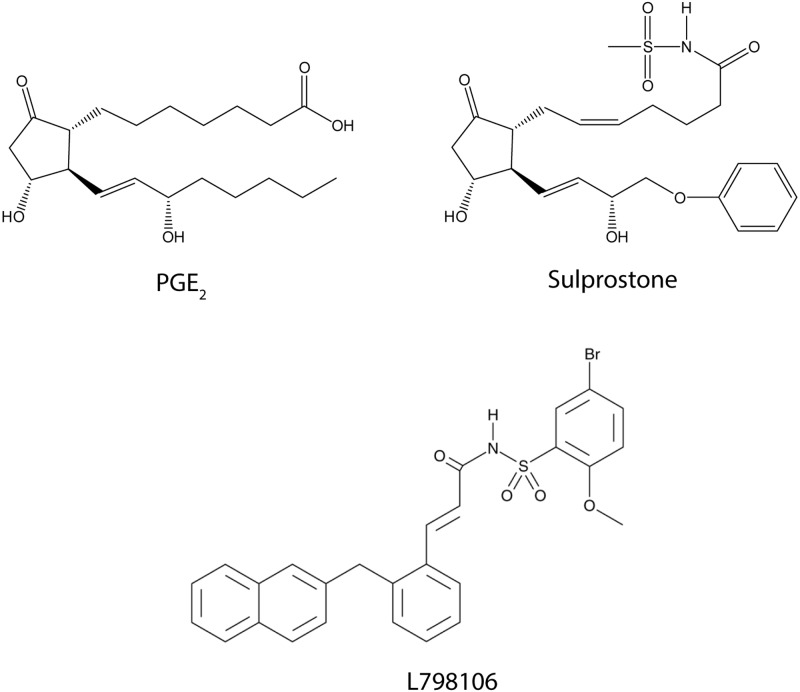
The structure of agonists and antagonists of EP3 receptors.

**FIGURE 4:**
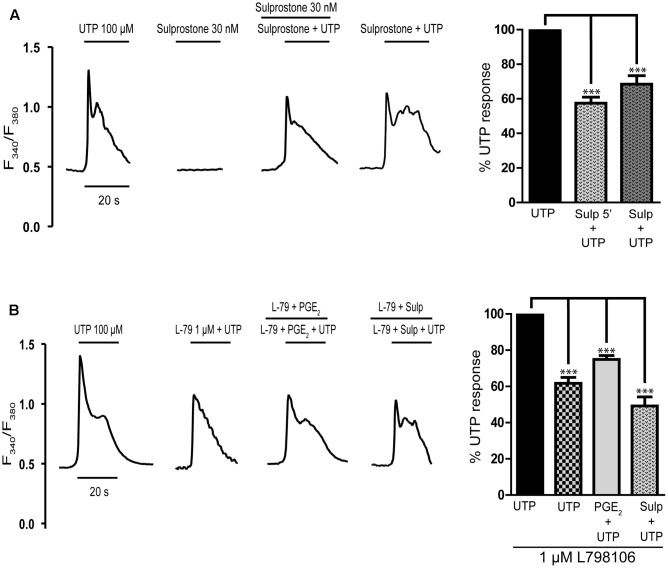
Effects of EP3 receptor ligands on UTP calcium responses in rat cerebellar astrocytes. **(A)** Sulprostone dampened the UTP calcium responses. Astrocytes were stimulated with UTP (100 μM), 30 nM Sulprostone or both these agonists, and the calcium responses were recorded. Where indicated, the cells were pre-incubated with sulprostone for 5 min before UTP stimulation. **(B)** Effect of L798106, an antagonist of EP3 receptors, on the calcium responses elicited by UTP and on PGE_2_ modulation. Where indicated, cells were pre-incubated with L798106 (1 μM) or with L798106 and PGE_2_/sulprostone, prior to stimulation with the nucleotide. Typical calcium traces are shown, and the graphs represent the quantification of the responses obtained as described in **Figure 1** and presented as the means ± SEM (*n* = 227 cells from three different cultures). ^∗∗∗^*p* < 0.0001 according to one way analysis of variance and Dunnett’s post-test.

Having excluded the participation of EP1 and EP2/EP4 receptors in the inhibitory effect of PGE_2_ on the calcium responses to UTP, we assessed the involvement of EP3 receptors in this effect (**Figure 3**). The EP3 agonist sulprostone is a synthetic analog of PGE_2_ that is resistant to metabolism and it reproduced the effect of PGE_2_ (**Figure 4A**). Stimulation with UTP (100 μM) after preincubation with sulprostone (30 nM, 5 min) dampened the calcium responses, which reached 57.44% of the control responses. Likewise, co-stimulation with sulprostone and UTP resulted in calcium responses that reached 61.71% of the control UTP responses. These findings supported the involvement of EP3 receptors in this phenomenon. In fact, EP3 receptors were detected in Western blots, although assays carried out with an EP3 receptor antagonist, L798106 ((2E)-*N*-[(5-bromo-2-methoxyphenyl) sulfonyl]-3-[2-(2-naphthalenylmethyl) phenyl]-2-propenamide), proved inconclusive. This antagonist did not modify the basal calcium levels but it did depress the calcium responses to UTP by 40% (**Figure 4B**). Moreover, pretreatment of astrocytes with L798106 prior to their stimulation with PGE_2_ or sulprostone did not prevent their inhibitory effects. Thus, despite the detection of EP3 receptors in Western blots (**Figure 8**), their involvement in the regulatory effect of PGE_2_ remained unclear. Indeed, we suggest that L798106 might be interacting with P2Y receptors given its similar structure to non-selective antagonists of nucleotide receptors, such as PPADS or suramin (Ho et al., 1995; Communi et al., 1996; Jimenez et al., 2000).

### PGE_2_ Impairs the Cell Migration Induced by UTP in Cerebellar Astrocytes

**FIGURE 5:**
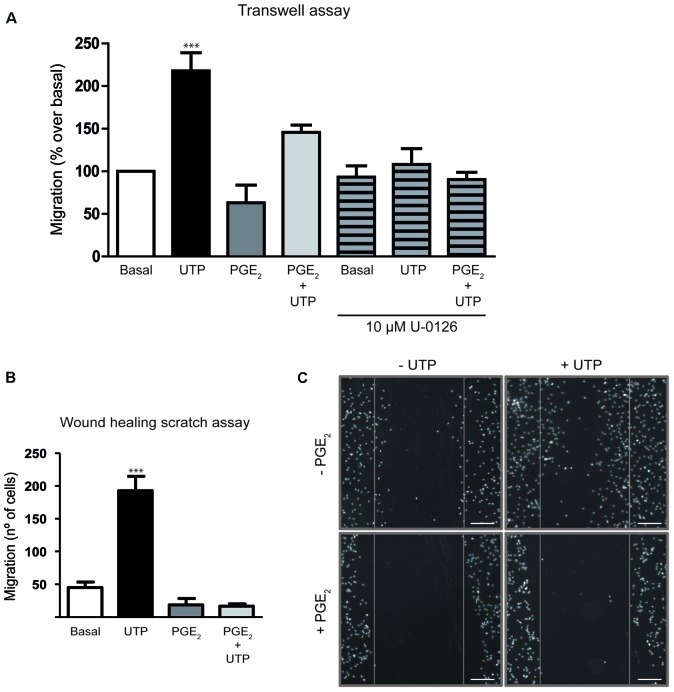
Prostaglandin E_2_ impairs rat cerebellar astrocyte migration. **(A)** The capacity of astrocytes to migrate in the Transwell assay. Cells were added to the upper chamber of transwell plates with inserts (8 μm porous), with UTP (100 μM) in the lower chamber as a chemoattractant. The cells were preincubated in the presence or absence of PGE_2_ (1 μM) for 5 min prior to being placed on the insert. After 18 h, cell migration was determined as described in Section “Materials and Methods.” Similar experiments were carried out with cells pretreated for 20 min with U0126 (10 μM). **(B)** *In vitro* wound-healing assay. Wound-healing experiments were carried out after inducing a standard wound in an astrocyte monolayer, washing with PBS and analyzing cell replenishment after 24 h. Where indicated the cells were preincubated with PGE_2_ (1 μM) for 5 min prior to UTP (100 μM) stimulation. **(C)** Representative images of cell nuclei revealed with DAPI staining in a wound-healing experiment (Magnification 10X). Scale bar = 200 μm. The results are expressed as the means ± SEM of migrating (%) or invading cells (number of cells) with respect to that obtained in control conditions, in the absence of any effectors. ^∗∗∗^*p* < 0.0001 according to one way analysis of variance and Dunnett’s post-test.

We next examined whether PGE_2_ modulated the migration of cerebellar astrocytes induced by UTP, as reported in macrophages and fibroblasts (Pimentel-Santillana et al., 2014). In both assays used, transwell migration and wound-healing, PGE_2_ attenuated the migration of astrocytes induced by UTP (**Figure 5**). When UTP (100 μM) was added to the lower transwell chamber of cell culture dishes containing membrane inserts, transmembrane chemotactic migration of astrocytes was enhanced (**Figure 5A**). However, preincubation of cells with PGE_2_ (1 μM, 5 min) prior to their introduction into the upper chamber diminished their migration under control conditions and that induced by the nucleotide by 45%. UTP-induced chemotaxis was also prevented by U0126 (**Figure 5A**), an inhibitor of MEK, which indicated that ERK activation was required for cell migration. In wound-healing migration assays, PGE_2_ completely prevented cell migration in control conditions (without additional exogenous nucleotide), as well as that induced by the nucleotide (**Figure 5B**). In this case, prostaglandin was included in the fresh medium added after the cells were washed to remove cell debris.

### Characterization of the Intracellular Mechanism Used by PGE_2_ to Modulate the Responses to UTP

**FIGURE 6:**
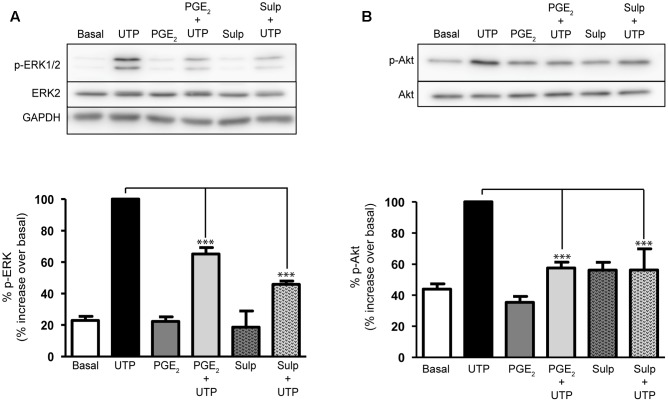
Prostaglandin E_2_ modulation on ERK and Akt activation elicited by UTP. Astrocytes were stimulated for 5 min with UTP (100 μM), PGE_2_ (1 μM) or Sulprostone (30 nM), and the phosphorylated and non-phosphorylated forms of ERK1/2 **(A)** and **(B)** Akt proteins present in total lysates were assessed in Western blots. Where indicated the cells were preincubated with PGE_2_ or sulprostone for 5 min prior to stimulation with the nucleotide. The blots shown are representative of independent experiments and GAPDH was used as an internal loading control. The values are the means ± SEM from 24 independent experiments performed on different cultures and the data is presented relative to the phosphorylation elicited by UTP (100 μM) in each individual experiment. ^∗∗∗^*p* < 0.0001.

The impact of PGE_2_ on the intracellular signaling cascades triggered by UTP was analyzed to further study the mechanism through which PGE_2_ modulates UTP responses. The ERK1/2 and PI3-kinase/Akt proteins are known to play relevant roles in nucleotide signaling and cell migration (Brambilla et al., 2002; Jimenez et al., 2002; Neary et al., 2004). Indeed, stimulation of astrocytes with UTP (100 μM) induced a remarkable increase in the phosphorylation of ERK1/2 (a fourfold to eightfold increase over basal levels) and of the Akt protein (a twofold increase over basal levels: **Figure 6**). Pretreatment of astrocytes with PGE_2_ (1 μM) reduced the ERK and Akt phosphorylation induced by UTP 35 and 40%, respectively. Stimulating astrocytes with PGE_2_ alone had no significant effect on ERK phosphorylation but it did dampen the phosphorylation of Akt. The inhibitory effects of PGE_2_ were also reproduced by sulprostone. Preincubation with the prostanoids was not necessary, and similar effects were observed after co-stimulation with PGE_2_/sulprostone and the nucleotide (results not shown). The extent of inhibition is consistent with the changes in the calcium responses observed.

### EGFR Is Required for PGE_2_ to Modulate UTP-Induced ERK1/2 and PI3-K/Akt Activation

**FIGURE 7:**
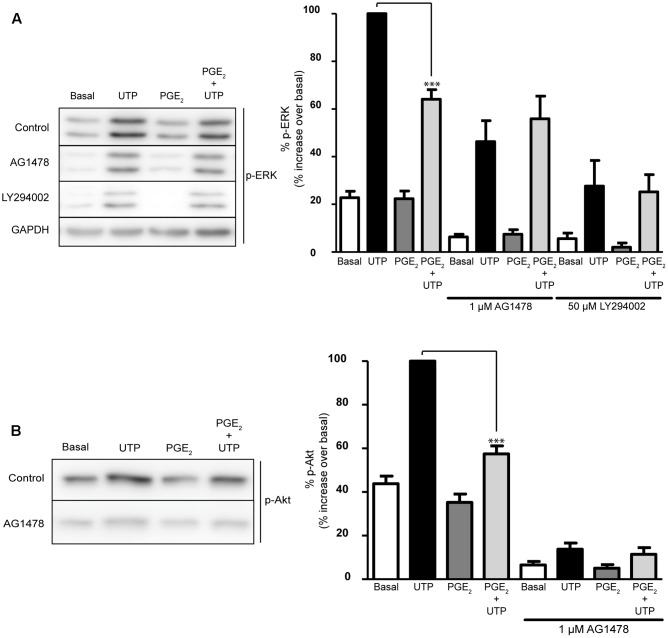
Modulation of UTP-induced ERK1/2 and PI3-K/Akt activation by PGE_2_ is dependent on EGFR and PI3K activation. Astrocytes were preincubated for 20 min in the presence or absence of AG1478 (1 μM) or LY294002 (50 μM) prior to stimulation with UTP (100 μM) for 5 min, and the p-ERK1/2 **(A)** and p-Akt **(B)** in the total lysates was detected in Western blots. Where indicated, the cells were treated with PGE_2_ (1 μM) for 5 min prior to stimulation with the nucleotide. The blots shown are representative of independent experiments and GAPDH was used as an internal loading control. GAPDH blot corresponding to that obtained in the presence of AG1478. The values are the means ± SEM from five independent experiments performed on different cultures and the results are presented as the phosphorylation relative to that elicited by UTP (100 μM) in each individual experiment. ^∗∗∗^*p* < 0.0001.

The EGF receptor plays a key role in the intracellular signaling cascades triggered by GPCRs, including P2Y_2_ receptors (Liu et al., 2004). To determine whether EGFR activation was required for the activation of ERK and/or Akt by the nucleotide, or for the modulatory effect of PGE_2_, the influence of an EGFR tyrosine kinase inhibitor was tested, AG1478. Interestingly, pretreatment of cells with AG1478 (1 μM) for 20 min reduced the basal phosphorylation of the two kinases, although it did not affect the UTP-induced ERK and Akt phosphorylation (**Figure 7**). Nevertheless, the EGFR inhibitor impaired the regulatory effect of PGE_2_, indicating that EGFR transactivation was required for the effect of the prostaglandin. Moreover, inhibition of PI3K with LY294002 (50 μM, 20 min) not only reduced the basal phosphorylation of ERK but it also prevented the regulatory effect of PGE_2_, although like the EGFR inhibitor it did not affect UTP-induced ERK phosphorylation. Thus, PGE_2_ could regulate ERK and Akt activation by a mechanism dependent on the transactivation of the EGFR and PI3K kinase, which would also support the involvement of EP3 receptors. To clearly demonstrate the possible implication of these receptors, more comprehensive studies will be necessary that take into account the different splice variants of these receptors described.

### Modulation of UTP-Induced ERK1/2 and PI3-K/Akt Activation by PGE_2_ in Astrocytes Activated with LPS

**FIGURE 8:**
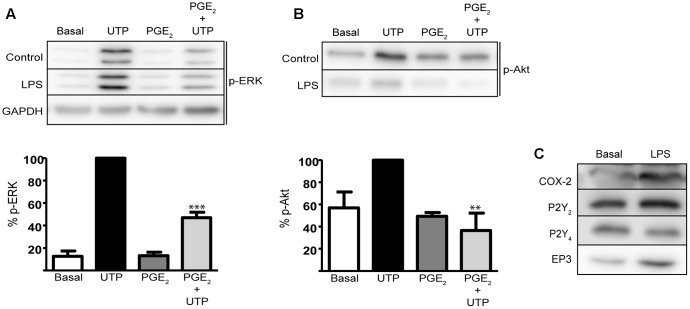
Prostaglandin E_2_ modulates UTP-induced ERK1/2 and PI3-K/Akt activation in astrocytes activated with LPS. Astrocytes were incubated in the presence or absence of LPS (10 ng/ml) for 24 h prior to analyzing the modulatory effect of PGE_2_ on the increase in p-ERK1/2 **(A)** and p-Akt **(B)** induced by UTP (see **Figure 6**). The Western blots are representative of independent experiments and GAPDH was used as an internal loading control. The values are the means ± SEM from three independent experiments performed on different cultures and the results are expressed relative to the UTP response in each individual experiment. **(C)** LPS induced COX-2 and EP3 receptor expression in rat cerebellar astrocytes. The presence of COX-2, P2Y2R, P2Y4R, and EP3R proteins was detected in Western blots of total cell lysates. ^∗∗∗^*p* < 0.0001, ^∗∗^*p* < 0.001.

To determine whether the modulation of PGE_2_ might be relevant to neuroinflammation and pathological conditions, we investigated the cross-talk between PGE_2_ and nucleotides in astrocytes treated with LPS, a toll-like receptor (TLR) ligand. Pretreatment with LPS (10 ng/mL, 24 h) did not affect either UTP-induced ERK phosphorylation or the modulatory effect of PGE_2_ (**Figure 8A**). UTP-induced ERK activation was higher (a 7.98-fold increase over unstimulated cells) than that observed in untreated cells (4.5-fold increase over basal levels) in the same experimental conditions, while PGE_2_ decreased UTP-induced ERK activation by 55%. Pretreatment of astrocytes with LPS notably increased the basal levels of the Akt phosphorylation and it diminished UTP-induced Akt phosphorylation by 55% (**Figure 8B**). In addition, PGE_2_ prevented Akt activation induced by the nucleotide. LPS induced strong expression of COX-2 (twofold over basal) but it did not significantly modify the levels of P2Y_2_/P2Y_4_ receptors in cerebellar astrocytes. Note that as reported previously, the P2Y_2_ receptor is more abundant in these cells than the P2Y_4_ receptor. Nevertheless, LPS also increased the levels of EP3 receptors (twofold to threefold increase over basal levels) (**Figure 8C**).

## Discussion

**FIGURE 9:**
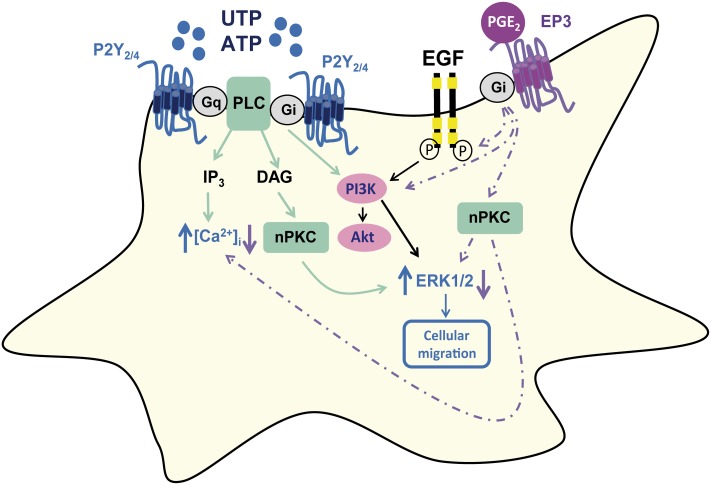
Schematic representation of PGE_2_ modulation of UTP signaling in rat cerebellar astrocytes. PGE_2_ dampened the calcium responses induced by UTP stimulation of P2Y_2_/P2Y_4_, as well as reducing the UTP-induced ERK and Akt activation, and cell migration. The effect of PGE_2_ was dependent on EP3 receptor interactions, and it involved EGFR transactivation and PI3K activity, although the involvement of nPKC could be not excluded.

The current study was carried out on cultured rat cerebellar astrocytes, a good model to address the regulation of nucleotide P2Y receptors. We previously characterized the function of the different P2Y receptors present in individual astrocytes by microfluorimetry using fura-2 (Jimenez et al., 2000). We found that all astrocytes in our cultures were sensitive to ATP/UTP stimulation, which provoked a metabotropic calcium response. Furthermore, the metabotropic ATP calcium responses mediated by P2Y_2_/P2Y_4_ receptors were modulated by other agonists of the purinergic system, such as adenosine, the final product of extracellular nucleotide degradation, and the dinucleotide Ap_5_A, which is also stored in secretory vesicles and that has potentiating activity (Jimenez et al., 1999, 2002; Delicado et al., 2005). Similar effects were observed with signals that activated Gs-coupled receptors and with EGF. Here we identified another mechanism that regulates the metabotropic calcium responses to ATP and we demonstrate that PGE_2_ inhibits UTP signaling in cerebellar astrocytes, not only impairing intracellular calcium mobilization but also dampening ERK activation, activation of the PI3K/Akt axis, and impairing cell migration (**Figure 9**).

The effect of PGE_2_ on UTP driven calcium responses was also evaluated at the single cell level. The fact that PGE_2_ modulation was observed in all the cells tested suggests universal and homogenous expression of the P2Y_2_/P2Y_4_ receptors of UTP in these glial cells, although it also indicated that a specific target for PGE_2_ might be present in these cells. In fact, although, most of the studies were performed on cell populations, the 40% inhibition displayed by PGE_2_ was similar to that obtained in individual cells. Although glial cells had more P2Y_2_ and P2Y_4_ receptors than murine macrophages, the inhibitory effect of PGE_2_ on UTP calcium responses in astrocytes was weaker than that found in macrophages and fibroblasts, in which it accounted for 60–70% inhibition (Traves et al., 2013). Nevertheless, the data gathered here indicate astrocytes are another cell type involved in tissue remodeling, in which PGE_2_ negatively modulates UTP and/or ATP responses, in addition to macrophages and fibroblasts.

Considering that co-stimulation of astrocytes with PGE_2_ and UTP reproduced the inhibitory effect observed in cells previously exposed to PGE_2_, and intracellular calcium mobilization is one of the first steps of the intracellular cascades triggered by GPCRs, the target/s of PGE_2_ might be located at the plasma membrane. This raises the question as to whether the activity of PGE_2_ is mediated by EP receptors, and what might be the intracellular mechanism and the final target of PGE_2_ used to impair the responses to UTP. Moreover, it raises interest in the physiological implications of the cross-talk between PGE_2_ and P2Y nucleotide receptors in these glial cells. Focusing on the possible interaction of PGE_2_ with EP receptors, all four subtypes of EP receptors (EP1–EP4) have been described in the brain, and they are present in neurons and glial cells (Sang et al., 2005; Di Cesare et al., 2006; Kunori et al., 2011). The presence of functional EP1 receptors in cerebellar astrocytes was ruled out as PGE_2_ and its analogs did not induce calcium responses. These findings contrast to those of astrocytes isolated from other brain sites, such as the hippocampus and cortex (Bezzi et al., 1998; Di Cesare et al., 2006). Rat cortical astrocytes were sensitive to PGE_2_ (1 μM) challenges, EP1, EP2/EP4, and EP3 receptors evoking calcium responses heterogeneously in the astrocyte population. Moreover, in cortical astrocytes, calcium responses mediated by EP2/EP4 receptors were reproduced by isoproterenol, a β-agonist, and forskolin, a direct adenylate cyclase activator (Di Cesare et al., 2006). In cerebellar astrocytes neither isoproterenol nor forskolin triggered calcium responses (Jimenez et al., 1999), whereas extracellular signals coupled to adenylate cyclase stimulation strongly potentiated metabotropic ATP calcium responses. This potentiation was parallel to but independent of cAMP accumulation, suggesting the involvement of βγ subunits released after Gs stimulation. The data reported here indicated that PGE_2_ was unable to potentiate UTP calcium responses, suggesting that EP2/EP4 receptors were neither present nor involved in the inhibitory effect of PGE_2_, although their presence cannot been ruled out. PGE2 has been shown to evoke calcium responses and potentiated glutamate-induced calcium signaling in astrocytes from the OVLT (organum vasculosum laminae terminals) and MnPO (median preoptic nucleus), contributing to the manifestation of fever (Simm et al., 2016).

The effects of PGE_2_ in cerebellar astrocytes could be mediated by EP3 receptors, as they were detected by immunobloting and sulprostone reproduced all the modulatory roles of the prostaglandin (**Figure 9**). Sulprostone was even more potent than PGE_2_ in reducing UTP-induced ERK activation, and this EP3 agonist slightly increased ERK phosphorylation in unstimulated cells and prevented UTP-induced Akt phosphorylation. The expression of EP3 receptors increased following exposure to LPS, consistent with the increase in these receptors in astrocytes and microglia after ischemic stroke (Han et al., 2016). Nevertheless, the actions of the two agonists assayed, the physiological and the synthetic PGE_2_ analog, were not reversed by the EP3 receptor antagonist, L798106, which depressed UTP calcium responses. However, L79829 might also interact with P2 nucleotide receptors, acting as a non-competitive antagonist. The interaction of PGE_2_ with EP3 receptors could induce Gi/o protein recruitment, a mechanism possibly responsible for the inhibition of UTP calcium responses. In cerebellar astrocytes, metabotropic calcium responses and ERK activation induced by the nucleotide were sensitive to PTX (Carrasquero, 2008), and PGE_2_ abolished the PTX-sensitive component of UTP calcium responses. Thus, an effect on G protein recruitment could also account for the reduction of ERK activation by PGE_2_ and sulprostone described previously. To identify the target and the pathway involved in the modulation of UTP responses by PGE_2_, we extended the signaling studies to the PI3K/Akt axis that plays a key role in the nervous system, in development, neuroprotection, proliferation and cancer (Ortega et al., 2008, 2009; Fransson et al., 2013). As expected, UTP induced Akt phosphorylation, agreeing with data reported in rat cortical astrocytes (Jacques-Silva et al., 2004). Moreover, PGE_2_ decreased both the basal phosphorylation of Akt and that Akt induced by the nucleotide.

The PI3K axis can be activated by Gi/o-proteins and/or the transactivation of tyrosine kinase receptors that are also present in cortical and cerebellar astrocytes, including EGF and NGF receptors (Lenz et al., 2001; Jimenez et al., 2002; Delicado et al., 2005). The treatment of astrocytes with AG1478 reduces the basal phosphorylation of ERKs and Akt, revealing that EGFR transactivation lies upstream of these kinases. This may not be surprising as the cells are cultured with FCS, although this did not affect the ERK phosphorylation induced by UTP, which was even higher than that obtained in control conditions, nor Akt phosphorylation. AG1478 strongly impeded the modulatory action of PGE_2_, suggesting that transactivation of EGFR is one step of the intracellular mechanism triggered by the PGE_2_, opening new roles for the EGFR in rat cerebellar astrocytes. It was clearly demonstrated that EGFR participates in the inhibition of UTP responses displayed by PGE_2_ when high nucleotide concentrations accompany PGE_2_ in the extracellular milieu. However, when the nucleotide levels diminish EGFR can contribute to the maintenance of the actions of nucleotides, as EGF potentiated previously ineffective UTP/ATP concentrations.

Subsequent studies with LY294002 revealed that PI3K inhibition has similar effects to EGFR inhibition, which would make it interesting to explore the targets of Akt. More than 50 substrates have been identified for this protein, the phosphorylation of which might positively or negatively modulate their functions, alter their subcellular localization, or modify their stability (Liao and Hung, 2010). One of the most relevant substrates is glycogen synthase kinase 3 (GSK3), which regulates cell metabolism and survival in the nervous system (Bhat et al., 2004). Unpublished results (not shown) indicate that GSK3 is also phosphorylated in astrocytes after UTP stimulation to a similar extent as Akt, although PGE_2_ only reduced UTP-induced phosphorylation by 20%. GSK3 is a well-known negative regulator of the enzyme responsible for the glycogen synthesis, glycogen synthase. Glycogen is mainly synthesized and accumulated in astrocytes, and it is mobilized and transformed into lactate to be supplied for neurons in situations of hypoxia or upon demand (Suzuki et al., 2011).

PI3K might also interact with some PKC isoforms regulating their activation. Some PKC isoforms present in cerebellar astrocytes can interact with this kinase, such as PKC-α, -δ, and -ζ identified in cortical astrocytes (Chen and Chen, 1996). In neuroblastoma cells, the atypical PKC-ζ dampened Akt phosphorylation, thereby providing a negative feedback loop to regulate P2X7 expression (Gomez-Villafuertes et al., 2015). In cerebellar astrocytes, it could be responsible for decreasing GSK3 phosphorylation, although we did not find evidence of an involvement of PKC-ζ in this phenomenon. The involvement of other PKC isoforms cannot be ruled out and nPKC could be responsible for the phosphorylation of P2Y_2_/P2Y_4_ receptors, which could be internalized to dampen the calcium responses. Moreover, DAG production/nPKC activation may be required for nucleotides to drive ERK activity in these glial cells, particularly as UTP-P2Y receptors were sensitive to feedback inhibition by PKC (Jimenez et al., 2000; Flores et al., 2005). In fact, in astrocytes in which nPKC was down-regulated by prolonged exposure to phorbol dibutyrate (PDBu) the UTP calcium responses were stronger than those obtained in control cells (results not shown). In macrophages and fibroblasts, the effect of PGE_2_ was dependent on PKC and PKD activation, although it was also independent of EP receptors (Traves et al., 2013; Pimentel-Santillana et al., 2014).

### Physiological Implications

This study aimed to explore the cross-talk between PGE_2_ and UTP-sensitive P2Y receptors in cerebellar astrocytes. We found that PGE_2_ negatively modulated UTP responses, as reported previously in macrophages and fibroblasts. However, there were two differences inasmuch as the effect of PGE_2_ appeared to be dependent on EP3 receptors and also, it was observed in LPS treated cells. In macrophages the interaction took place in thioglycollate-elicited and alternative macrophages (M2), but not in macrophages activated with the endotoxin (M1). While the metabolic changes that occur during macrophage activation and the transition from M1 to M2 phenotype have been largely explored (Rodriguez-Prados et al., 2010; Traves et al., 2012), this is not the case for astrocytes activated with LPS. Nevertheless, macrophages and astrocytes share some metabolic features and they are constitutively glycolytic (Bolanos, 2016). Hence, it will be very interesting to investigate the metabolic implications of the cross-talk reported in cerebellar astrocytes, both in control conditions and in LPS-treated astrocytes, and the intracellular targets of the endotoxin. The negative effects of PGE_2_ on UTP signaling could contribute to limit excessive astrogliosis, which could represent part of the adaptive mechanism to neuroinflammation. Several studies have indicated that both P2Y and P2X receptors are upregulated in the astrogliosis process involving ERK and Akt activation (Franke et al., 2001; Neary et al., 2005; Franke and Illes, 2014). We are developing studies to determine whether P2Y_2_/P2Y_4_ receptors are upregulated after prolonged treatment with the endotoxin and whether they can be down-regulated by PGE_2_.

## Author Contributions

ED, LB, and RP-S designed experiments; LP-H, JG-R, MQ, and SG-R performed experiments; ED, RP-S, and MM-P wrote the manuscript; LP-H, JG-R, and MQ made illustrations and generated figures; MM-P and ED directed the project.

## Conflict of Interest Statement

The authors declare that the research was conducted in the absence of any commercial or financial relationships that could be construed as a potential conflict of interest.

## Abbreviations

[Ca^2+^]_i_: intracellular free calcium concentration
DMEM: Dulbecco’s modified Eagle’s medium
EGF: epidermal growth factor
EGFR: epidermal growth factor receptor
ERK: extracellular signal regulated kinases
FCS: fetal calf serum
GPCRs: G protein-coupled receptors
GAPDH: glyceraldehyde-3-phosphate dehydrogenase
LPS: lipopolysaccharide
MEK: mitogen extracellular activated kinases
NGF: nerve growth factor
PI-3 kinase: phosphatidylinositol-4,5-bisphosphate 3-kinase
nPKC: novel protein kinase C
PKD: protein kinase D
PPADS: pyridoxal phosphate-6-azophenyl-2′,4′-disulfonic acid
PGs: prostaglandins
PGE_2_: prostaglandin E_2_
